# The efficacy and safety of Femoston combined with Baoqing granules for kidney Yin deficiency in perimenopausal women: a randomized, double-blind, placebo-controlled trial

**DOI:** 10.3389/fendo.2026.1791905

**Published:** 2026-04-15

**Authors:** Chun Li, Hong-yan Lei, Ruo-lan Wang, Jing-bo Zhang, Zhi-man Huang, Hong-ping Shen, Yu-lin Luo, Tao Ye, Yong-zhou Wang, Yuan Yuan

**Affiliations:** 1 Center of Evidence-based Medicine, The Affiliated Traditional Chinese Medicine Hospital, Southwest Medical University, Luzhou, Sichuan, China; 2 Department of Gynecology, The Affiliated Traditional Chinese Medicine Hospital, Southwest Medical University, Luzhou, Sichuan, China; 3 Department of Medicine, Ziyang College of Dental Technology, Ziyang, Sichuan, China; 4 School of Public Health, Southwest Medical University, Luzhou, Sichuan, China; 5 Department of Traditional Chinese Medicine, The Third Affiliated Hospital of Chengdu Medical College, Chengdu, Sichuan, China

**Keywords:** Baoqing granules, efficacy, perimenopausal syndrome, randomized clinical trial, vasomotor symptoms

## Abstract

**Background:**

Perimenopausal symptoms affect most women and often substantially impair their quality of life, whereas conventional estrogen therapy alone does not fully address clinical needs. Baoqing granules (BQG), derived from *Zuo Gui Wan* in *Jingyue’s Complete Works*, were modified to target kidney Yin deficiency and have shown promising clinical efficacy, although robust randomized evidence remains limited.

**Methods:**

The trial was registered with the Chinese Clinical Trial Registry (ChiCTR2300073338; http://www.chictr.org.cn). In this randomized, double-blind, placebo-controlled clinical trial, eligible perimenopausal women received either Femoston plus placebo or Femoston plus BQG for four weeks, followed by a four-week follow-up. The primary outcomes were the Modified Kupperman Menopausal Index (Modified KMI) and traditional Chinese medicine (TCM) syndrome scores. Secondary outcomes included the Menopause-Specific Quality of Life Questionnaire (MENQOL), Pittsburgh Sleep Quality Index (PSQI), Self-Rating Anxiety Scale (SAS), Self-Rating Depression Scale (SDS), and serum levels of sex hormones and lipids. Safety was evaluated through hepatic and renal function tests and adverse-event monitoring.

**Results:**

Both groups showed significant improvements from baseline; however, participants in the BQG group experienced greater short-term improvements that persisted at week 8 in Modified KMI, TCM syndrome scores, MENQOL, and PSQI, while differences in SAS and SDS became significant only at follow-up. Hormone and lipid profiles showed no overall between-group differences, although exploratory subgroup analyses suggested higher estradiol and lower follicle-stimulating hormone levels in postmenopausal women receiving BQG. Liver and kidney functions remained within normal ranges, and only one mild adverse event was reported.

**Conclusion:**

In this 8-week study, BQG as an adjunct to Femoston was associated with additional short-term improvements in multidimensional perimenopausal symptoms, with acceptable tolerability. These findings support the potential role of BQG as a complementary therapy; however, longer-term studies are needed to confirm the durability of benefit and long-term safety.

**Clinical Trial Registration:**

http://www.chictr.org.cn, identifier ChiCTR2300073338.

## 1 Introduction

Menopause is a natural physiological transition resulting from the decline of ovarian follicular function, with approximately 90% of women experiencing it between the ages of 45 and 56 years (1). The period encompassing the late reproductive stage through the early postmenopausal phase is collectively termed perimenopause (2). During this transition, fluctuating ovarian activity and hormonal instability can lead to a spectrum of endocrine-related symptoms collectively referred to as perimenopausal syndrome (PMPS). These manifestations include vasomotor symptoms (hot flashes, night sweats), psychological and cognitive disturbances (anxiety, depression, memory decline), urogenital discomfort (vaginal dryness, urinary frequency), sleep disorders, and metabolic or musculoskeletal complaints such as joint pain and osteoporosis (3). Epidemiological data indicate that 50–75% of women experience vasomotor symptoms lasting for more than seven years, significantly impairing quality of life (1). Moreover, approximately 10% of women develop depressive symptoms, particularly those with a prior history of depression (4). PMPS also increases the risk of chronic diseases such as cardiovascular disorders and osteoporosis, posing a substantial public health burden.

Menopausal hormone therapy, also known as hormone replacement therapy (HRT), remains the standard treatment for alleviating menopausal symptoms. HRT effectively mitigates vasomotor symptoms and helps prevent osteoporosis and, to some extent, cardiovascular disease (5, 6). Femoston, a representative HRT formulation containing estradiol (E2) and dydrogesterone, provides estrogen replacement while protecting the endometrium and is widely used in clinical practice. However, the long-term use of HRT is limited by safety concerns. Numerous studies have linked HRT to an increased risk of breast, endometrial, and ovarian cancers, venous thromboembolism, and cardiovascular events (7–13). Furthermore, some women continue to experience residual symptoms—such as hot flashes, mood disturbances, and sleep disorders—despite treatment, while adverse effects including breast tenderness and nausea can reduce adherence and compromise efficacy (3, 14, 15). Non-hormonal pharmacologic agents such as paroxetine, venlafaxine, escitalopram, and gabapentin can alleviate vasomotor and psychological symptoms, but side effects including drowsiness, weight gain, and fatigue limit their clinical utility (13). These limitations underscore the need for safe, effective, and multi-target complementary interventions to improve the quality of life of perimenopausal women.

In traditional Chinese medicine (TCM), the pathogenesis of menopausal transition was first described in *The Yellow Emperor’s Inner Canon*, which attributes it to a “decline in kidney Qi and deficiency of kidney Yin”. Contemporary TCM consensus likewise identifies kidney Yin deficiency as the predominant syndrome pattern among perimenopausal women, accounting for approximately 80–90% of cases (16). Accordingly, nourishing kidney Yin and calming the liver and mind are regarded as the core therapeutic principles. In recent years, herbal therapies have gained increasing attention for their natural origin, favorable safety profile, and good patient tolerance, and are being explored as adjunctive or alternative approaches for managing menopausal symptoms (3).

Baoqing granules (BQG) is a compound herbal formula derived from the classical prescription Zuogui Wan, modified to better address kidney Yin deficiency. The formula consists of *Rehmanniae Radix Praeparata*, *Dioscoreae Rhizoma*, *Corni Fructus*, *Cistanches Herba*, *Cuscutae Semen*, *Lycii Fructus*, *Schisandra chinensis Fructus*, and *Os Draconis*. According to TCM theory, BQG nourishes kidney Yin, replenishes essence, and calms the mind. Pharmacological and clinical studies provide evidence supporting its potential therapeutic effects. For example, herbal formulas containing *Rehmannia glutinosa* as the principal component have been reported to alleviate perimenopausal symptoms in breast cancer patients (17); catalpol, a major active compound of *Rehmannia*, exhibits antidepressant activity via the SIRT1 pathway (18). Extracts from *Dioscorea* species have been shown to improve menopausal psychological symptoms (19), while *Semen Cuscutae* may regulate bone metabolism through the OPG/RANKL signaling pathway (20). *Lycium barbarum* polysaccharides and glycopeptides also possess neuroprotective and metabolic regulatory properties (21–23). Collectively, these findings provide pharmacological support for the potential application of BQG in managing menopausal symptoms.

Preclinical and preliminary clinical studies further suggest that BQG may alleviate perimenopausal symptoms and improve quality of life by modulating sex hormone levels and regulating apoptotic pathways through the downregulation of Bax and the upregulation of Bcl-2 and VEGF (24–26). BQG is produced in accordance with Chinese national pharmaceutical standards and has been approved as an in-hospital preparation in several medical institutions.

Nevertheless, current evidence regarding BQG remains largely empirical, with few rigorously designed randomized controlled trials. Therefore, this study was conducted as a randomized, double-blind, placebo-controlled clinical trial to systematically evaluate the efficacy and safety of BQG combined with Femoston in perimenopausal women with kidney Yin deficiency.

## 2 Methods

### 2.1 Materials

All crude herbal materials were procured from a certified supplier of traditional Chinese medicinal decoction pieces, and their quality met the standards specified in the Pharmacopoeia of the People’s Republic of China (2020 edition). BQG were manufactured by a licensed pharmaceutical institution using standardized procedures, including soaking, extraction, precipitation, concentration, and decoction, to produce a uniform paste extract.

The placebo was formulated by incorporating 3% BQG extract into inert excipients with suitable coloring and flavoring agents to ensure an identical appearance, odor, taste, and dosage form to BQG. Because Chinese herbal medicine granules often have distinctive organoleptic characteristics, complete sensory matching using fully inert excipients alone can be difficult (27). Methodological studies have suggested that inclusion of a very small proportion of the original herbal extract may improve organoleptic similarity and help preserve blinding (28). Therefore, 3% BQG extract was included in the placebo for sensory simulation rather than for therapeutic exposure. Both BQG and placebo underwent comprehensive quality control analyses, including thin-layer chromatography identification, particle size measurement, moisture content determination, solubility testing, fill-weight uniformity, and microbial limit assays. All results conformed to the relevant pharmaceutical quality standards.

Nutritional composition analyses verified compliance with national food safety regulations, and no *Escherichia coli*, molds, yeasts, or *Salmonella* were detected. All products were stored in a cool, well-ventilated environment, uniformly packaged by the manufacturer, and certified by the Sichuan Provincial Food and Drug Administration (Certificate No. SZBZ20081021-11[z]). Detailed quality inspection reports are provided in the **Supplementary Material**.

The HRT administered in this study was a fixed-dose combination of E2 and dydrogesterone (Femoston^®^, Abbott Biologicals B.V., Netherlands), with each tablet containing 1 mg of E2 and 10 mg of dydrogesterone.

### 2.2 Study design and ethical approval

This study was a randomized, double-blind, placebo-controlled clinical trial designed to evaluate the efficacy and safety of BQG combined with Femoston in perimenopausal women with kidney Yin deficiency syndrome. Participants were recruited from the Department of Gynecology, Affiliated Hospital of Traditional Chinese Medicine, Southwest Medical University, between January 1, 2023, and May 31, 2024.

**Figure 1:**
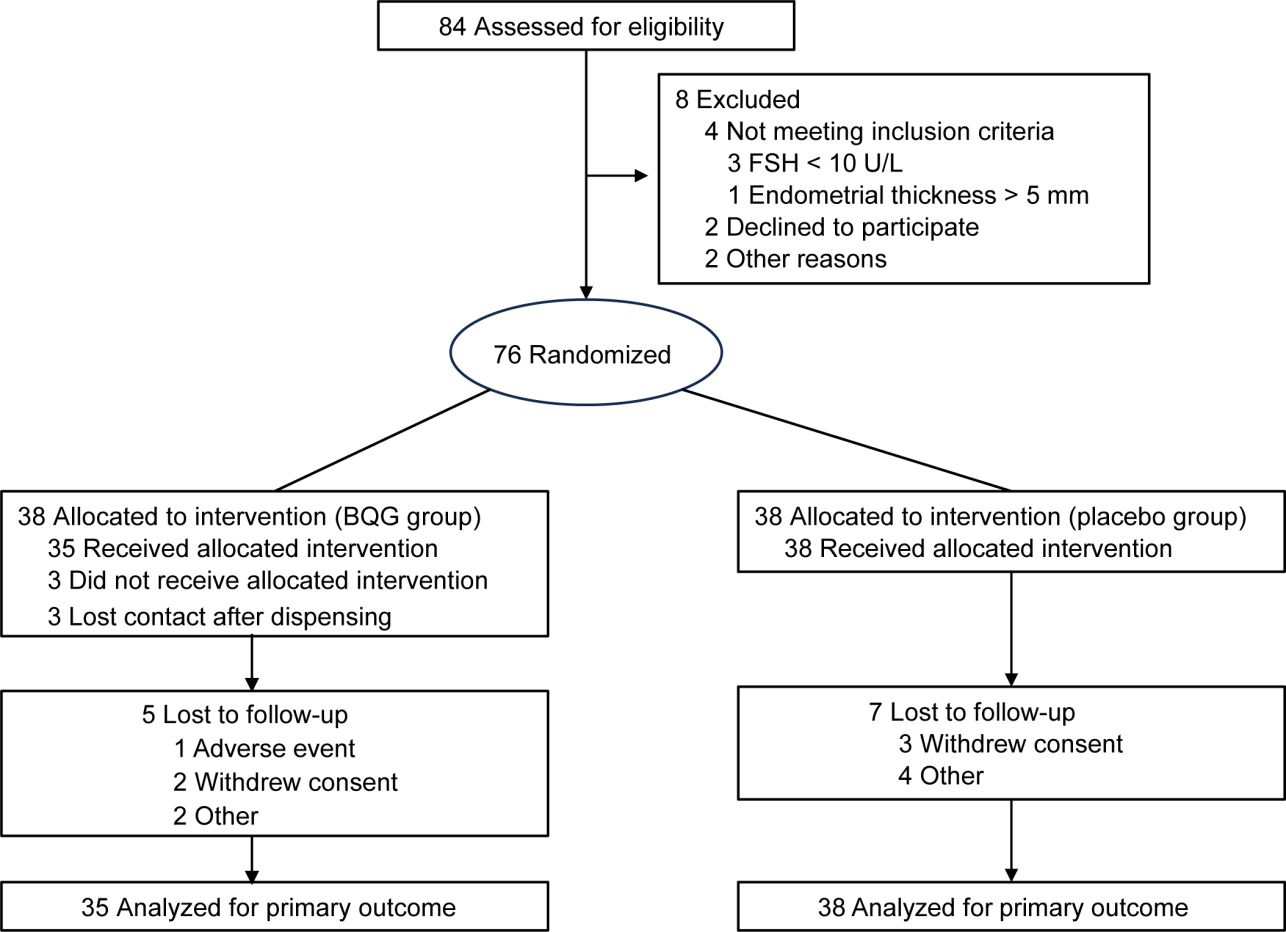
CONSORT 2025 flow diagram of participant enrollment, allocation, follow-up, and analysis.

The trial was conducted in accordance with the principles of the Declaration of Helsinki. The study protocol and statistical analysis plan were reviewed and approved by the Institutional Review Board of the Affiliated Hospital of Traditional Chinese Medicine, Southwest Medical University (Approval No. KY2023022-FS01). The trial was registered with the Chinese Clinical Trial Registry (ChiCTR2300073338), which is recognized by the International Committee of Medical Journal Editors. The full study protocol is publicly accessible via the registry website (http://www.chictr.org.cn). All participants provided written informed consent prior to enrollment. The overall study flow is illustrated in **Figure 1**, and detailed reporting followed the CONSORT 2025 checklist (**Supplementary Material**).

### 2.3 Participants

#### 2.3.1 Inclusion criteria

Eligible participants were women aged 40–55 years who met the diagnostic criteria for PMPS according to the STRAW + 10 reproductive aging staging system and *Gynecology* (9th edition, Wang Zhehua, 2024) (2, 29). Laboratory examinations confirmed serum follicle-stimulating hormone (FSH) levels greater than 10 IU/L, accompanied by at least two of the following symptoms: menstrual irregularity, vasomotor disturbances, autonomic dysfunction, or neuropsychiatric manifestations. Transvaginal ultrasonography demonstrated an endometrial thickness of less than 5 mm (single layer) (30).

In addition to the biomedical criteria, participants were required to meet the TCM diagnostic criteria for kidney Yin deficiency, as defined in the Standards for Diagnosis and Curative Effect Evaluation of TCM Syndromes (ZY/T 001.1–94, State Administration of Traditional Chinese Medicine). The diagnosis required the presence of at least one primary symptom (e.g., menstrual disorder, hot flashes with aversion to cold, or mood fluctuation) and at least two secondary symptoms (e.g., soreness and weakness of the waist and knees, fatigue, dizziness, tinnitus, irritability, insomnia with excessive dreaming, or vaginal dryness). Supportive diagnostic signs included a red tongue with little or yellowish coating and a thin or rapid pulse.

Participants were included only if they had a Modified Kupperman Menopausal Index (Modified KMI) score greater than 15 (31), no contraindications to E2/dydrogesterone therapy, and had provided written informed consent prior to enrollment.

#### 2.3.2 Exclusion criteria

Participants were excluded if they had known hypersensitivity to any study medication; participated in another clinical trial or received hormonal or investigational therapy within the previous three months; or were currently using medications or supplements targeting perimenopausal symptoms. Women with severe cardiovascular, hematologic, hepatic, renal, or other major systemic diseases were excluded, as were those with psychiatric disorders or a history of substance abuse.

Additional exclusion criteria included a history of bilateral oophorectomy (except for benign lesions before the age of 40), ovarian tumors, premature ovarian failure, malignancies such as breast or endometrial cancer or meningioma, hyperthyroidism, porphyria, or severe spinal disorders. Participants with hepatic or renal insufficiency, unexplained vaginal bleeding, untreated progressive endometrial hyperplasia, venous thromboembolism, or recent arterial thrombotic events were also excluded. Investigators reserved the right to exclude any individual deemed unsuitable for participation for medical or ethical reasons.

#### 2.3.3 Withdrawal criteria

Participants were withdrawn from the study in the event of serious adverse events, medical or ethical indications requiring discontinuation, poor treatment adherence, or the use of prohibited medications. Participants could also withdraw voluntarily due to unsatisfactory efficacy, intolerance to adverse effects, preference for alternative treatments, or personal reasons.

### 2.4 Sample size, randomization, and blinding

The sample size was determined based on a one-sided α of 0.025 and a statistical power of 80% (β = 0.20). A previous systematic review reported that estrogen-based therapies reduce vasomotor symptoms by approximately 75% compared with placebo (95% CI, 64.3–82.3) (32). Given that the primary outcomes of this study were composite measures—the Modified KMI and TCM syndrome score—and that the population comprised both premenopausal and postmenopausal women with a relatively short treatment duration, a conservative estimation was adopted to avoid overestimating the control response rate and underpowering the trial.

The effective response rate in the control group was assumed to be 70%, with an anticipated 24% improvement in the treatment group. Using a 1:1 allocation ratio and a two-sample comparison of proportions, the minimum required sample size was calculated to be 37 participants per group (total n = 74). Allowing for a 15% expected dropout rate, the final target enrollment was set at 87 participants. All calculations were performed using standard statistical software (PASS, version 15; NCSS, USA), and the formula is provided below:


$$n=\frac{{\left(Z_{1-\frac{\alpha }{2}}+Z_{1-\beta }\right)}^{2}}{{\left(p_{1}-p_{2}\right)}^{2}}[\frac{p_{1}(1-p_{1})}{k}+p_{2}(1-p_{2})]$$

Randomization was conducted by an independent statistician using a computer-generated random sequence with a 1:1 allocation ratio. Allocation codes were sealed in sequentially numbered, opaque envelopes and kept by an independent custodian. Investigators accessed the envelopes sequentially only after confirming participant eligibility, thereby ensuring allocation concealment and the independence of randomization.

A double-blind design was maintained throughout the study. Blinding was implemented at two hierarchical levels: (1) anonymized group codes (Group A = control, Group B = BQG) managed solely by the independent statistician; and (2) intervention assignments (control or BQG) managed by a designated drug administrator. The placebo was identical to BQG in appearance, smell, taste, and dosage form. Throughout the trial, investigators, participants, and outcome assessors remained blinded to treatment allocation to ensure objectivity and minimize bias.

### 2.5 Intervention and follow-up

A standardized case report form was developed by the research team to ensure consistent and systematic documentation of all clinical and follow-up data. All participants received standard HRT consisting of one tablet of Femoston administered orally once daily. On this basis, participants in the treatment group additionally received BQG, whereas those in the control group received a matching placebo. Both BQG and placebo were taken orally at a dose of 10 g three times daily for 4 weeks.

Follow-up assessments were conducted immediately after the 4-week intervention and again 4 weeks later. The 4-week treatment duration was selected to assess early symptomatic response based on prior clinical experience at our institution. In addition, the primary and secondary symptom-based outcome measures used in this study are considered suitable for detecting clinical change, and prior studies have demonstrated that menopausal symptom improvement can be observed within short-term assessment windows (33). Each evaluation included symptom scoring, laboratory examinations, and safety assessments. At the end of the study, all remaining study medications were collected and disposed of by the designated drug administrator in accordance with institutional standard operating procedures.

The treatment regimen was maintained consistently throughout the trial until completion or withdrawal according to predefined criteria. Prior to treatment initiation, investigators provided participants with detailed explanations of study procedures, medication instructions, and participation restrictions. The use of any medication that could interfere with study outcomes was prohibited, although maintenance therapies for stable chronic conditions were permitted. In cases of early withdrawal, the reasons were documented in detail, and investigators endeavored to complete final evaluations to ensure data integrity.

### 2.6 Outcomes

All participants were evaluated at baseline, week 4 (end of intervention), and week 8 (follow-up). Assessments included primary outcomes, secondary outcomes, and safety parameters.

The study used two prespecified co-primary outcomes: the Modified KMI and the TCM syndrome score (31, 34). The Modified KMI was selected to evaluate the overall severity of menopausal symptoms using a widely accepted clinical scale, whereas the TCM syndrome score was included to assess changes in kidney Yin deficiency–related manifestations in accordance with the TCM diagnostic framework used for participant selection. Because perimenopausal syndrome is multidimensional in nature, these co-primary outcomes were intended to capture complementary aspects of treatment response from both the conventional symptom-based perspective and the TCM syndrome-based perspective.

The Modified KMI, one of the most widely used tools for assessing menopausal symptoms, has been validated in Chinese populations (31). It comprises 13 items covering hot flashes and sweating, paresthesia, insomnia, nervousness, depression, dizziness, fatigue, musculoskeletal pain, headache, palpitations, thyroid-related symptoms, sexual complaints, and urinary tract infections. The total score ranges from 0 to 39 and is classified as follows: <6, asymptomatic; 7–15, mild; 16–30, moderate; and >30, severe.

The TCM syndrome score was developed according to the Standards for Diagnosis and Curative Effect Evaluation of TCM Syndromes and is widely used to assess symptom improvement in TCM-based interventions (35). The scoring system includes characteristic manifestations of kidney Yin deficiency, such as night sweats, palpitations, restlessness, spontaneous sweating, dizziness, tinnitus, hot flashes, insomnia with vivid dreams, menstrual irregularities, feverish sensations in the palms and soles, memory decline, and soreness of the waist and knees. Each item is rated on a 1–5 scale, with higher scores indicating greater symptom severity, yielding a total score range of 11 to 55. In this study, the total score was used to reflect overall syndrome severity and its change over time.

Therapeutic efficacy was quantified using an efficacy index (EI), calculated as: EI (%) = (score before treatment−score after treatment)​/score before treatment × 100. Efficacy was categorized as follows: cured (EI ≥ 90%), markedly effective (70–89%), effective (30–69%), and ineffective (< 30%).

The secondary outcomes included quality of life, sleep quality, emotional status, and laboratory parameters. Because these domains reflect interrelated but non-identical aspects of perimenopausal symptomatology, the selected measures were used to provide a complementary assessment of treatment response. Although some overlap exists among them, each was included to capture a distinct aspect of clinical outcome.

The Menopause-Specific Quality of Life Questionnaire (MENQOL) contains 29 items across four domains—vasomotor, psychosocial, physical, and sexual. Each item is rated on a seven-point Likert scale, with lower scores indicating better quality of life (36).

Sleep quality was assessed using the Pittsburgh Sleep Quality Index (PSQI), a 19-item self-reported questionnaire evaluating seven components: subjective sleep quality, sleep latency, sleep duration, habitual sleep efficiency, sleep disturbances, use of sleep medication, and daytime dysfunction. Total scores range from 0 to 21, with higher scores reflecting poorer sleep quality (37).

Emotional status was assessed using Zung’s Self-Rating Anxiety Scale (SAS) and Self-Rating Depression Scale (SDS), each comprising 20 items evaluating somatic and cognitive symptoms of anxiety or depression. Higher scores indicate greater symptom severity (38, 39).

Laboratory indicators included serum levels of E2, FSH, and luteinizing hormone (LH), as well as lipid profiles—serum triglycerides (TG) and total cholesterol (TC)—to evaluate ovarian function and metabolic status.

### 2.7 Safety assessment

Safety assessments included the monitoring of vital signs (body temperature, pulse rate, blood pressure, and respiratory rate) and the documentation of all adverse events throughout the study period. Liver and kidney function tests—including alanine aminotransferase (ALT), aspartate aminotransferase (AST), and creatinine (CR)—were performed at baseline, after the 4-week intervention, and at the 8-week follow-up to evaluate treatment safety. All adverse events were recorded in detail, including their onset, duration, severity, and potential relationship to the study medication, and managed as clinically indicated. Continuous safety monitoring was implemented to ensure the timely identification and appropriate handling of any adverse reactions.

### 2.8 Statistical analysis

The primary efficacy analysis was conducted using the full analysis set (FAS) in accordance with the intention-to-treat (ITT) principle. Missing data were imputed based on variable type. For clinical rating scales (e.g., symptom and quality-of-life scores), the worst-case imputation method was applied as a conservative approach under the ITT principle to minimize the risk of overestimating treatment effects in symptom-based outcomes. For laboratory parameters, mean substitution was used because these variables were primarily supportive or safety-related measures and the extent of missingness was limited. Baseline values were defined as the final recorded measurements obtained prior to the first administration of the study drug. The Modified KMI and TCM syndrome score were prespecified as co-primary efficacy endpoints. To account for potential multiplicity associated with the use of two primary endpoints, a Bonferroni-corrected threshold (*p* < 0.017) was additionally used as a conservative reference for between-group comparisons. A per-protocol (PP) analysis was additionally performed as a sensitivity analysis to assess the robustness of the primary efficacy findings. The safety analysis included all participants who received at least one dose of the study medication and completed at least one post-baseline efficacy evaluation.

Categorical variables were summarized as frequencies and percentages, and between-group differences were analyzed using the chi-square test or Fisher’s exact test, as appropriate. The normality of continuous variables was assessed using the Shapiro–Wilk test, and the homogeneity of variances was evaluated with Levene’s test. Normally distributed data were expressed as mean ± standard deviation (SD), whereas non-normally distributed data were presented as median (interquartile range, IQR). For normally distributed variables with equal variances, between-group comparisons were performed using independent-sample t-tests, and within-group comparisons were conducted using paired t-tests. For non-normally distributed data, the Mann–Whitney U test and Wilcoxon signed-rank test were applied for between-group and within-group analyses, respectively.

To evaluate treatment effects over time, a mixed-design analysis of variance (ANOVA) was performed, incorporating group (between-subject factor), time (within-subject factor), and the group × time interaction. Effect sizes for repeated-measures ANOVA were additionally reported as partial η² to facilitate interpretation of the magnitude of the observed effects. When the assumption of sphericity was violated, Greenhouse–Geisser corrections were applied to adjust the degrees of freedom. The Friedman test was used to analyze repeated-measures data that were not normally distributed. Exploratory subgroup analyses by menopausal status were conducted to assess potential hormonal differences. Correlations between continuous variables were examined using Pearson’s correlation coefficient for normally distributed data and Spearman’s rank correlation coefficient for non-normally distributed data.

Safety analyses were descriptive and included summaries of the incidence, type, and severity of adverse events, each reviewed on a case-by-case basis. All statistical analyses were conducted using SPSS version 25.0 (IBM Corp., Armonk, NY, USA), and graphical visualizations were generated with GraphPad Prism version 10.0 (GraphPad Software, San Diego, CA, USA). All tests were two-tailed, and a *p*-value < 0.05 was considered statistically significant.

## 3 Results

### 3.1 Patient characteristics at baseline

The participant flow is shown in **Figure 1**. A total of 84 women provided written informed consent and underwent eligibility screening. Eight participants were excluded: four did not meet the inclusion criteria (three with FSH < 10 U/L and one with endometrial thickness > 5 mm), two declined participation, and two were excluded for other reasons. Consequently, 76 eligible participants were randomly assigned to the two study groups.

In the BQG group, 35 participants received the allocated intervention, while three did not initiate treatment after drug dispensing. During the study, five participants were lost to follow-up (one due to an adverse event, two who withdrew consent, and two for other reasons), resulting in 35 participants being included in the primary efficacy analysis according to the ITT principle.

In the control group, all 38 participants received the allocated intervention. Seven participants were lost to follow-up (three withdrew consent and four for other reasons), and all 38 were included in the primary efficacy analysis in accordance with the ITT principle.

Baseline demographic and clinical characteristics are summarized in **Table 1**. No statistically significant differences were observed between the two groups in any baseline parameter, indicating good comparability at study entry.

**Table 1:** Demographic and baseline characteristics of the participants.

Characteristics	BQG group (n = 35)	Control group (n = 38)	*p*-value
Age (year, mean ± SD)	47.71 ± 4.19	47.71 ± 3.72	0.997
Height (cm, mean ± SD)	156.91 ± 4.36	158.29 ± 5.11	0.222
Weight (kg, mean ± SD)	57.26 ± 6.63	57.13 ± 7.80	0.941
BMI (kg/m^2^, mean ± SD)	23.23 ± 2.35	22.93 ± 3.12	0.644
Systolic blood pressure (mmHg, mean ± SD)	117.71 ± 8.17	118.89 ± 6.73	0.501
Diastolic blood pressure (mmHg, mean ± SD)	75.09 ± 5.39	75.87 ± 5.62	0.546
History of cervical cyst, n (%)	13 (37.1%)	7 (18.4%)	0.073^a^
Multiparous (≥ 2 births), n (%)	19 (54.3%)	15 (39.5%)	0.205^a^
Postmenopausal, n (%)	15 (42.9%)	16 (42.1%)	0.948^a^
TG (mmol/L, median [IQR])	1.42 (0.95,2.61)	1.29 (0.90,1.74)	0.911^b^
TC (mmol/L, mean ± SD)	5.08 ± 0.84	5.21 ± 0.86	0.498
CR (μmol/L, mean ± SD)	62.43 ± 10.08	60.35 ± 10.71	0.396
ALT (U/L, mean ± SD)	21.78 ± 15.96	18.22 ± 10.23	0.256
AST (U/L, mean ± SD)	22.39 ± 9.71	20.16 ± 5.21	0.220

Data are presented as n (%), mean ± SD, or median (IQR), as appropriate. ^a^*p*-value calculated using the chi-square test. ^b^*p*-value calculated using the Mann–Whitney U test.

### 3.2 Primary outcomes

Improvements in menopausal symptoms, assessed using the Modified KMI and the TCM syndrome score, are summarized below.

**Figure 2:**
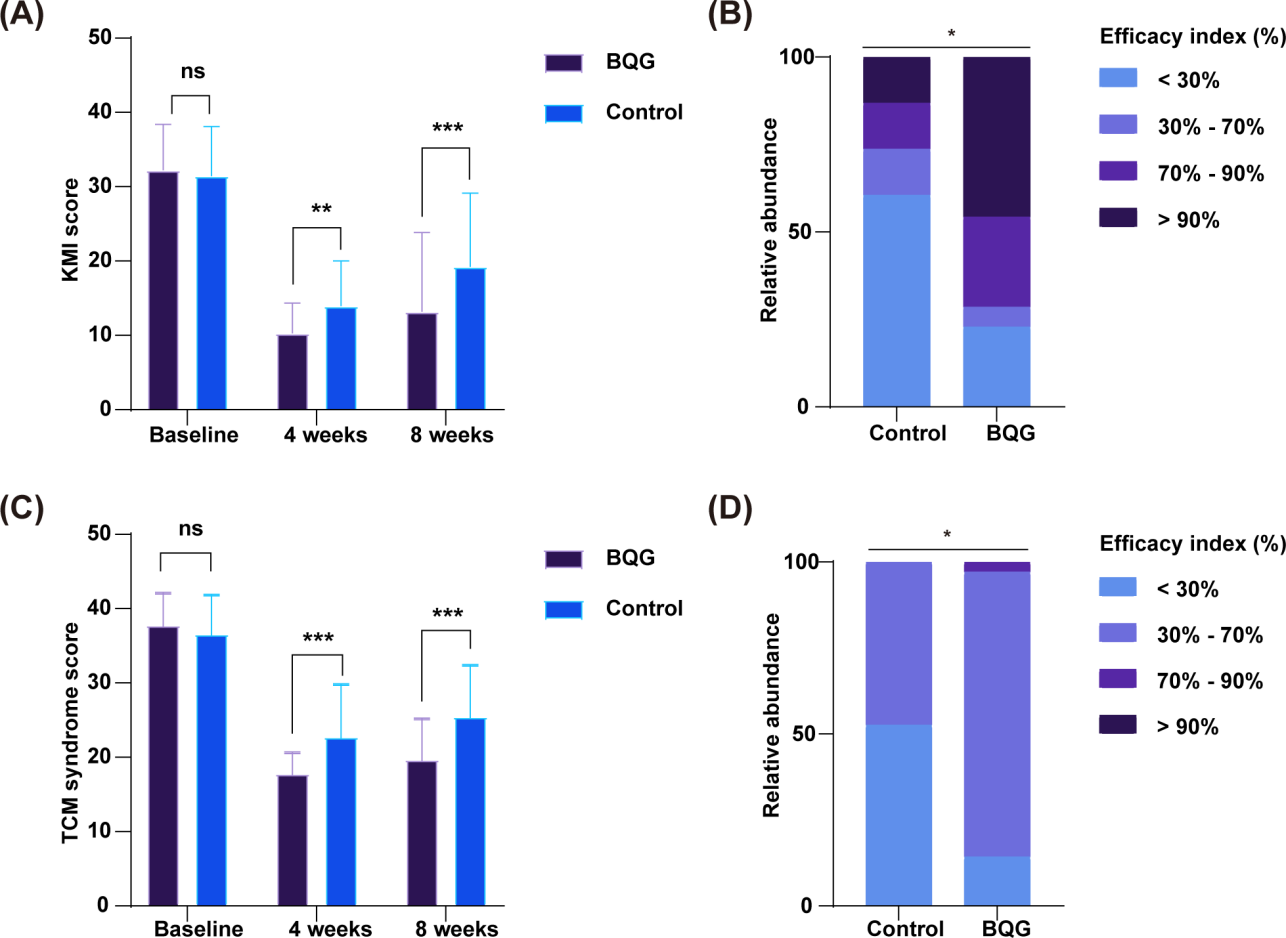
Changes in the modified KMI and TCM syndrome scores in the BQG and control groups. **(A)** Changes in Modified KMI scores; **(B)** EI analysis based on the Modified KMI; **(C)** Changes in TCM syndrome scores; **(D)** EI analysis based on the TCM syndrome score. ns, not significant; *p < 0.05; **p < 0.01; ***p < 0.001.

As shown in **Table 2** and **Figure 2A**, baseline KMI scores were comparable between groups (32.23 ± 6.18 vs. 31.45 ± 6.64, *p* = 0.605), indicating good balance at study entry. After 4 weeks of treatment, both groups exhibited significant reductions in KMI scores; however, the decrease was significantly greater in the BQG group (10.63 ± 5.44 vs. 13.29 ± 6.03, *p* = 0.004). At week 8, both groups showed a slight rebound, yet KMI scores remained significantly lower in the BQG group compared with the control group (13.17 ± 10.68 vs. 19.24 ± 9.90, *p* = 0.014). Repeated-measures ANOVA revealed significant main effects of time (*F* = 160.513, *p* < 0.001, partial η² = 0.69), group (*F* = 6.372, *p* = 0.014, partial η² = 0.08), and time × group interaction (*F* = 4.458, *p* = 0.022, partial η² = 0.06), suggesting that the combination therapy achieved a more pronounced improvement that remained observable at week 8. Clinically, this benefit was particularly evident in vasomotor symptoms such as hot flashes and night sweats.

**Table 2:** Comparisons of primary and secondary outcome scores between the BQG and control groups over time.

Groups	Variables	Time			*P*_baseline_	*P*_4wks_	*P*_8wks_	Time effect			Group effect			Time × group interaction effect		
		Baseline	4 weeks	8 weeks				*F*	*p*-value	Partial η²	*F*	*p*-value	Partial η²	*F*	*p*-value	Partial η²
BQG	KMI	32.23 ± 6.18	10.29 ± 4.06	13.17 ± 10.68	0.605	0.004	0.014	160.513	< 0.001	0.69	6.372	0.014	0.08	4.458	0.022	0.06
Control		31.45 ± 6.64	13.92 ± 6.11	19.24 ± 9.90												
BQG	TCM	37.57 ± 4.39	17.56 ± 2.97	19.47 ± 5.59	0.292	< 0.001	< 0.001	276.342	< 0.001	0.80	10.450	0.002	0.13	11.965	< 0.001	0.14
Control		36.34 ± 5.40	22.50 ± 7.19	25.23 ± 7.05												
BQG	MENQOL	62.80 ± 16.98	25.28 ± 14.42	25.15 ± 16.75	0.770	0.014	0.010	171.303	< 0.001	0.71	5.367	0.023	0.07	1.592	0.212	0.02
Control		64.03 ± 18.75	32.62 ± 10.19	33.39 ± 9.22												
BQG	PSQI	23.77 ± 6.07	9.20 ± 4.52	7.89 ± 4.68	0.467	0.015	< 0.001	203.794	< 0.001	0.74	6.214	0.015	0.08	10.399	< 0.001	0.13
Control		22.66 ± 6.87	11.82 ± 4.50	13.35 ± 4.89												
BQG	SAS	50.47 ± 6.22	38.36 ± 4.78	37.36 ± 6.38	0.949	0.053	0.001	116.880	< 0.001	0.62	6.335	0.014	0.08	4.254	0.016	0.06
Control		50.55 ± 5.50	40.78 ± 5.65	42.22 ± 6.11												
BQG	SDS	52.79 ± 7.00	42.46 ± 5.15	42.37 ± 4.65	0.879	0.206	0.049	76.572	< 0.001	0.52	1.673	0.200	0.02	1.166	0.312	0.02
Control		52.54 ± 7.10	44.10 ± 5.80	44.66 ± 5.05												

Data are presented as mean ± SD. *P_baseline_*, comparison between groups at baseline; *P_4wks_*, comparison between groups at 4 weeks; *P_8wks_*, comparison between groups at 8 weeks. Partial η² values are reported as effect size measures for repeated-measures ANOVA.

Similarly, baseline TCM syndrome scores did not differ significantly between groups (37.57 ± 4.39 vs. 36.34 ± 5.40, *p* = 0.292; **Table 2**; **Figure 2C**). After 4 weeks, the BQG group showed significantly lower scores than the control group (17.56 ± 2.97 vs. 22.50 ± 7.19, *p* < 0.001), and this superiority persisted at week 8 (19.47 ± 5.59 vs. 25.23 ± 7.05, *p* < 0.001). Repeated-measures ANOVA demonstrated significant main effects of time (*F* = 276.342, *p* < 0.001, partial η² = 0.80), group (*F* = 10.450, *p* = 0.002, partial η² = 0.13), and their interaction (*F* = 11.965, *p* < 0.001, partial η² = 0.14), confirming that participants in the BQG group experienced greater symptom relief over the 8-week study period, particularly in hallmark features of kidney Yin deficiency such as dry mouth, fatigue, and soreness or weakness of the lower back and knees. Importantly, the between-group differences for both co-primary endpoints remained statistically significant under the Bonferroni-corrected threshold of *p* < 0.017. Sensitivity analysis using the per-protocol population yielded results consistent with those of the primary ITT/FAS analysis for both co-primary endpoints, supporting the robustness of the main findings (**Supplementary Table 1**).

Further analysis based on the EI supported these findings. As illustrated in **Figures 2B, D**, the distribution of efficacy indices differed significantly between groups. The proportion of participants achieving an EI≥70%—corresponding to the “markedly effective” or “cured” categories—was substantially higher in the BQG group, whereas a larger proportion of participants in the control group fell within the EI < 30% (ineffective) range. These results indicate that BQG combined with Femoston provided superior overall efficacy and clinical benefit compared with Femoston alone.

### 3.3 Secondary outcomes

#### 3.3.1 Quality of life

**Figure 3:**
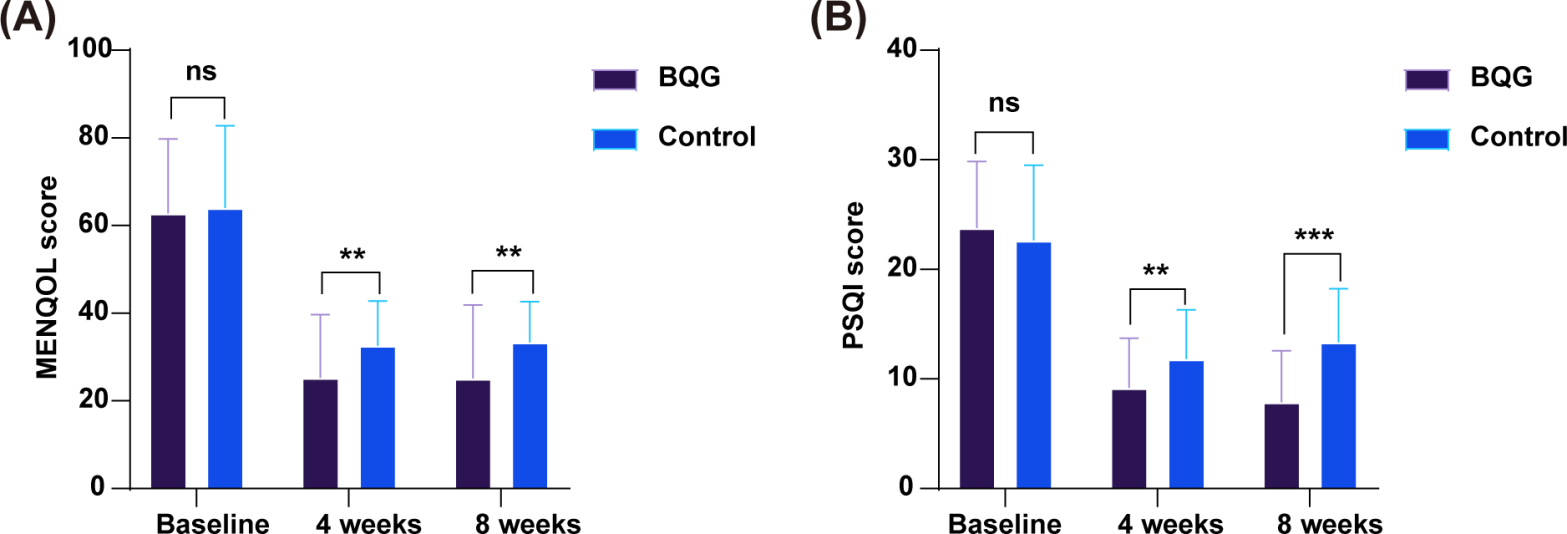
Changes in MENQOL and PSQI scores in the BQG and control groups. **(A)** Changes in MENQOL scores; **(B)** Changes in PSQI scores. ns, not significant; **p < 0.01; ***p < 0.001.

As shown in **Table 2** and **Figure 3**, there were no significant baseline differences between the two groups in MENQOL or PSQI scores (*p* = 0.770 and *p* = 0.467, respectively).

After treatment, total MENQOL scores decreased significantly in both groups, with greater improvement observed in the BQG group. At week 4 (25.28 ± 14.42 vs. 32.62 ± 10.19, *p* = 0.014) and week 8 (25.15 ± 16.75 vs. 33.39 ± 9.22, *p* = 0.010), MENQOL scores remained significantly lower in the BQG group than in the control group (**Figure 3A**). Repeated-measures ANOVA revealed significant main effects of time (*F* = 171.303, *p* < 0.001) and group (*F* = 5.367, *p* = 0.023), whereas the time × group interaction was not significant (*F* = 1.592, *p* = 0.212). These findings indicate that both treatments improved quality of life, with consistently greater reductions in MENQOL scores in the BQG group.

Similarly, PSQI scores declined significantly in both groups, with more pronounced improvements in the BQG group. The BQG group had significantly lower PSQI scores than the control group at both week 4 (9.20 ± 4.52 vs. 11.82 ± 4.50, *p* = 0.015) and week 8 (7.89 ± 4.68 vs. 13.35 ± 4.89, *p* < 0.001; **Figure 3B**). Repeated-measures ANOVA demonstrated significant effects of time (*F* = 203.794, *p* < 0.001), group (*F* = 6.214, *p* = 0.015), and time × group interaction (*F* = 10.399, *p* < 0.001), indicating that the combination therapy achieved more sustained and marked improvements in sleep quality.

#### 3.3.2 Psychological outcomes

**Figure 4:**
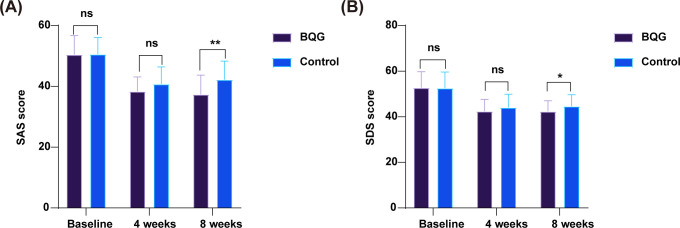
Changes in SAS and SDS scores in the BQG and control groups. **(A)** Changes in SAS score; **(B)** Changes in SDS score. ns, not significant; *p < 0.05; **p < 0.01.

As presented in **Table 2** and **Figure 4**, no significant differences were observed between the two groups in baseline SAS or SDS scores (*p* > 0.05 for both).

For anxiety, SAS scores progressively decreased in both groups throughout the study period. The between-group difference was not significant at week 4 (38.36 ± 4.78 vs. 40.78 ± 5.65, *p* = 0.053). By week 8, however, the BQG group exhibited a marked and statistically significant reduction compared with the control group (37.36 ± 6.38 vs. 42.22 ± 6.11, *p* = 0.001; **Figure 4A**). Repeated-measures ANOVA revealed significant main effects of time (*F* = 116.880, *p* < 0.001), group (*F* = 6.335, *p* = 0.014), and time × group interaction (*F* = 4.254, *p* = 0.016), indicating a consistent improvement in anxiety symptoms over time in both groups, with a more pronounced and sustained reduction in the BQG group.

For depression, SDS scores also declined significantly in both groups over time. The between-group difference remained nonsignificant at week 4 but reached statistical significance by week 8, with lower scores observed in the BQG group (42.37 ± 4.65 vs. 44.66 ± 5.05, *p* = 0.049; **Figure 4B**). Repeated-measures ANOVA showed a significant main effect of time (*F* = 76.572, *p* < 0.001), whereas the effects of group (*F* = 1.673, *p* = 0.200) and time × group interaction (*F* = 1.166, *p* = 0.312) were not significant, suggesting a general time-dependent improvement in depressive symptoms across both groups.

#### 3.3.3 Hormone and metabolic parameters

As shown in **Table 3**, no significant differences were observed between the two groups in serum E2, FSH, or LH levels at baseline, week 4, or week 8 (all *p* > 0.05). Given that the study population included both premenopausal and postmenopausal women, an exploratory subgroup analysis was performed to account for potential heterogeneity.

**Table 3:** Serum levels of E2, FSH, and LH in the BQG and control groups, including subgroup analyses of premenopausal and postmenopausal women.

Population	Variables	Baseline			4 weeks			8 weeks		
		BQG	Control	*p*-value	BQG	Control	*p*-value	BQG	Control	*p*-value
Overall										
	FSH (mIU/mL)	63.64 ± 30.61	66.37 ± 39.37	0.743	37.70 ± 16.24	38.73 ± 21.52	0.818	44.25 ± 27.82	53.42 ± 38.95	0.254
	LH (mIU/mL)	37.70 ± 16.24	38.73 ± 21.52	0.818	26.42 ± 15.97	28.05 ± 19.62	0.700	31.65 ± 17.32	31.98 ± 22.07	0.943
	E2 (pg/mL)	10.00 (5.00,40.14)	10.00 (5.00,22.75)	0.445	59.70 (13.20,95.80)	69.06 (12.55,173.50)	0.941	62.50 (5.00,86.50)	41.10 (5.00,69.06)	0.294
Subgroup analysis										
Postmenopausal	FSH (mIU/mL)	69.66 ± 35.60	86.61 ± 37.08	0.205	47.93 ± 34.84	50.15 ± 34.60	0.861	44.41 ± 30.86	73.22 ± 44.46	0.046
	LH (mIU/mL)	38.69 ± 11.63	51.95 ± 17.71	0.021	30.28 ± 16.04	36.28 ± 20.01	0.366	32.59 ± 19.12	43.65 ± 21.68	0.144
	E2 (pg/mL)	10.00 (5.00,20.93)	10.00 (5.00,10.00)	0.202	16.60 (5.00,94.83)	37.21 (9.72,79.37)	0.830	69.06 (25.90,95.00)	8.90 (5.00,62.22)	0.033
Premenopausal	FSH (mIU/mL)	59.11 ± 26.31	51.65 ± 34.77	0.441	28.89 ± 17.39	30.80 ± 24.90	0.777	44.41 ± 30.86	44.13 ± 26.13	0.977
	LH (mIU/mL)	36.95 ± 19.26	29.12 ± 19.02	0.193	23.53 ± 15.70	22.07 ± 17.42	0.777	30.95 ± 16.31	23.50 ± 18.52	0.176
	E2 (pg/mL)	12.55 (5.39,55.82)	17.12 (6.49,49.86)	0.869	77.25 (38.90,101.75)	103.05 (51.18,354.65)	0.236	47.80 (5.00,85.20)	69.06 (10.37,89.99)	0.648

Data are presented as mean ± SD, or median (IQR), as appropriate.

Among postmenopausal women, baseline FSH and E2 levels were comparable between groups, whereas LH levels were significantly higher in the control group than in the BQG group (51.95 ± 17.71 vs. 38.69 ± 11.63 mIU/mL, *p* = 0.021). By the end of follow-up, the BQG group exhibited a significantly lower FSH level (44.41 ± 30.86 vs. 73.22 ± 44.46 mIU/mL, *p* = 0.046) and a markedly higher median E2 concentration (69.06 pg/mL vs. 8.90 pg/mL, *p* = 0.029) compared with the control group, while LH levels remained comparable (*p* > 0.05), indicating that, although no overall differences were detected in the total population, BQG may exert a more pronounced endocrine regulatory effect in postmenopausal women.

In contrast, among premenopausal women, no significant between-group differences were found in FSH, LH, or E2 levels at any time point (all *p* > 0.05).

To further assess potential metabolic effects, serum TG and TC levels were evaluated. Both parameters remained within normal ranges throughout the study, with no significant between-group differences at most time points, except for a significant difference in TC levels at week 8 (*p* = 0.038; **Supplementary Table 2**).

### 3.4 Correlation analysis

To further clarify the interrelationships among the measured variables, correlation analyses were performed between the Modified KMI and TCM syndrome scores, as well as between these primary outcomes and secondary measures, including quality of life, sleep quality, and emotional status (**Table 4**).

**Table 4:** Correlation between primary outcomes and secondary indicators at 4 and 8 weeks.

Variables	4 weeks				8 weeks			
	KMI		TCM		KMI		TCM	
	BQG	Control	BQG	Control	BQG	Control	BQG	Control
KMI	/	/	0.634^**^	0.691^**^	/	/	0.787^**^	0.428^**^
TCM	0.634^**^	0.691^**^	/	/	0.787^**^	0.428^**^	/	/
SDS	0.082	0.291	0.160	0.521^**^	0.330	0.370^*^	0.380^*^	0.689^**^
SAS	0.363^*^	0.468^**^	0.554^**^	0.725^**^	0.426^*^	0.299	0.682^**^	0.738^**^
PSQI	0.558^**^	0.700^**^	0.376^*^	0.664^**^	0.814^**^	0.489^**^	0.839^**^	0.741^**^
MENQOL	0.620^**^	0.492^**^	0.421^*^	0.492^**^	0.744^**^	0.656^**^	0.767^**^	0.650^**^
FSH	0.351^*^	0.252	0.263	0.254	0.131	0.164	0.131	0.249
LH	0.438^**^	0.018	0.302	-0.019	0.116	0.164	0.098	0.123
E2	-0.265	-0.308	-0.279	-0.393^*^	0.228	0.043	0.000	-0.197

*p-value < 0.05; **p-value < 0.01.

A strong positive correlation was found between the Modified KMI and TCM syndrome scores, which progressively strengthened over time (4 weeks: *r* = 0.634, *p* < 0.01; 8 weeks: *r* = 0.787, *p* < 0.01).

At week 8, the Modified KMI scores showed significant positive correlations with MENQOL (*r* = 0.744, *p* < 0.01), PSQI (*r* = 0.814, *p* < 0.01), and SAS (*r* = 0.426, *p* < 0.05). Likewise, TCM syndrome scores were positively correlated with MENQOL (*r* = 0.767, *p* < 0.01), PSQI (*r* = 0.839, *p* < 0.01), SAS (*r* = 0.682, *p* < 0.01), and SDS (*r* = 0.380, *p* < 0.05). These findings indicate that the alleviation of menopausal symptoms was closely associated with concurrent improvements in quality of life, sleep, and emotional well-being, with the strength of these associations increasing over time.

In contrast, correlations between the primary outcome measures and serum sex hormone levels (E2, FSH, LH) were weak and not statistically significant, implying that the observed clinical improvements were unlikely to be primarily mediated by hormonal changes.

### 3.5 Safety and adverse events

No serious adverse events were reported during the study. A small number of participants experienced mild treatment-emergent adverse events. In the BQG group, one of 35 participants (2.9%) developed mild epigastric discomfort and nausea, which were tolerable but resulted in withdrawal from follow-up. Aside from this case, no clinically significant treatment-related events occurred in either group.

Laboratory assessments revealed no statistically significant differences between groups in liver or renal function parameters—including ALT, AST, and CR—at baseline, week 4, or week 8. All parameters remained within normal reference ranges throughout the study (**Table 5**).

**Table 5:** Liver and renal function parameters in the BQG and control groups over time.

Variables	BQG group	Control group	*P*_a_
ALT			
Baseline	21.78 ± 15.96	18.22 ± 10.23	0.256
4 weeks	18.45 ± 14.28	17.06 ± 7.54	0.599
8 weeks	19.68 ± 9.11	18.13 ± 8.00	0.442
*P*_b_	0.156	0.472	
*P*_c_	0.525	0.949	
AST			
Baseline	22.39 ± 9.71	20.16 ± 5.21	0.220
4 weeks	19.56 ± 8.08	19.56 ± 8.08	0.716
8 weeks	21.38 ± 7.20	20.35 ± 4.01	0.451
*P*_b_	0.061	0.187	
*P*_c_	0.656	0.783	
CR			
Baseline	62.43 ± 10.08	60.35 ± 10.71	0.396
4 weeks	61.56 ± 10.14	57.93 ± 8.31	0.098
8 weeks	60.37 ± 8.44	58.85 ± 8.92	0.458
*P*_b_	0.564	0.094	
*P*_c_	0.176	0.391	

Data are expressed as mean ± SD. *P*_a_ denotes the *p*-value for between-group comparisons; *P*_b_ denotes the *p*-value for within-group comparisons between baseline and week 4; *P*_c_ denotes the *p*-value for within-group comparisons between baseline and week 8.

Overall, BQG in combination with Femoston were well tolerated among perimenopausal women, with no evidence of hepatic or renal dysfunction or other safety concerns, thereby confirming the regimen’s favorable safety profile.

## 4 Discussion

This randomized, double-blind, placebo-controlled trial systematically evaluated the efficacy and safety of BQG as an adjunct to HRT with Femoston in perimenopausal women. The findings demonstrated that BQG significantly enhanced symptom improvement across multiple domains—including vasomotor, psychological, and sleep-related disturbances—while maintaining excellent tolerability.

Perimenopausal women frequently experience complex and fluctuating symptoms, and the efficacy of HRT in alleviating emotional and psychological disturbances remains controversial (40). Consequently, many women are turning to complementary and alternative medicine, with herbal therapies being the most widely used (41). Previous studies have reported that botanical products such as isoflavones, *Hypericum perforatum* (St. John’s wort), and red ginseng may relieve menopausal symptoms (42–44); however, clinical outcomes remain inconsistent (45). The therapeutic effects of herbal medicine depend on its bioactive constituents, dosage, duration, and individual variability (42). Compared with single-herb preparations, the multi-component formulation of BQG demonstrated broader short-term clinical benefits in this study.

Both the Modified KMI and TCM syndrome scores improved significantly more in the BQG group than in the control group, with partial persistence after discontinuation. The most notable improvements were observed in vasomotor symptoms (e.g., hot flashes, night sweats) and Yin-deficiency–related manifestations (e.g., dry mouth, fatigue, and weakness of the lower back and knees). Improvements in sleep and anxiety were accompanied by enhanced overall quality of life. Correlation analyses revealed strong associations between symptom relief and improvements in sleep, mood, and quality of life, but not with changes in serum hormone levels. These findings support the concept of “symptom–hormone dissociation”, suggesting that menopausal symptoms are not solely determined by circulating hormone concentrations (46, 47). Because fluctuations in FSH and E2 during the perimenopausal transition limit their reliability as indicators of symptom severity, patient-reported outcomes remain essential in evaluating treatment efficacy.

Recent neuroendocrine research has implicated hypothalamic KNDy neurons in thermoregulation and hot flash generation (48), while vasomotor instability may impair hippocampal and prefrontal cortical function, contributing to cognitive and emotional changes (49). The established efficacy of nonhormonal agents such as selective serotonin reuptake inhibitors, serotonin–norepinephrine reuptake inhibitors, and gabapentin in reducing vasomotor symptoms further supports the involvement of central neural and inflammatory mechanisms rather than purely endocrine pathways (50).

In exploratory subgroup analyses, postmenopausal women in the BQG group showed lower FSH and higher E2 levels at week 8. Given the negative feedback of E2 on FSH secretion, these findings may suggest a possible endocrine-modulating effect of BQG in this subgroup. Several active components of BQG, including schisandrin lignans, *Lycium barbarum* polysaccharides, and *Cistanche deserticola* extracts, have been reported to exhibit phytoestrogen-like activity (51–53), which may provide a biologically plausible explanation for this observation. However, these hormonal changes were modest and were identified only in exploratory subgroup analyses. Given the small subgroup sample size and the absence of significant between-group differences in the overall population, these findings should be interpreted with caution and regarded as hypothesis-generating rather than confirmatory. Further large-scale studies with predefined subgroup analyses and mechanistic investigations are warranted to clarify whether BQG has clinically meaningful endocrine effects in postmenopausal women.

Lipid parameters showed no consistent improvement, likely due to the relatively short treatment duration. Although TC levels differed significantly between groups at week 8, this change was not considered clinically meaningful and was likely attributable to data imputation or short-term variability rather than a true pharmacologic effect. Previous studies have shown that the lipid-modifying benefits of HRT generally require several months or even years to stabilize (54). A longer intervention period may therefore be necessary to evaluate potential metabolic effects more reliably.

Pharmacological evidence further supports the clinical findings of this study. The constituent herbs of BQG possess diverse biological activities that may act synergistically through multi-target and multi-pathway mechanisms. *Rehmannia Radix polysaccharides* and *Cuscuta species* have antidepressant and anxiolytic properties; *Cornus officinalis*, *Lycium barbarum*, and *Cistanches Herba* contribute to neuroprotection and improved sleep; *Dioscoreae Rhizoma* and *Lycium barbarum* enhance metabolism and physical endurance; and lignans and polysaccharides from *Schisandra chinensis Fructus* exhibit anti-fatigue, immunomodulatory, and hepatoprotective effects (55–63). In addition, mineral components such as *Os Draconis* contain carbon dots with anxiolytic activity, providing a modern mechanistic basis for the traditional TCM concept of “calming the mind” (64). Together, these pharmacological data offer a rational explanation for the multidimensional clinical benefits observed with BQG.

In terms of safety, only one participant experienced mild gastrointestinal discomfort, and no serious adverse reactions occurred. Liver and renal function parameters remained within normal ranges throughout the study, consistent with previous evidence on the safety of herbal medicines. These findings confirm that BQG combined with Femoston was well tolerated in perimenopausal women, with no evidence of hepatic, renal, or systemic toxicity.

In summary, this trial provides novel clinical evidence supporting the adjunctive use of BQG with standard hormone therapy to alleviate the multidimensional symptom burden of perimenopausal women. BQG demonstrated significant short-term efficacy and good tolerability. These findings highlight the potential clinical relevance of integrating evidence-based TCM into menopausal management. However, several limitations should be acknowledged. First, the relatively small sample size and single-center design may limit the generalizability of our findings. Second, participants were enrolled based on the TCM diagnosis of kidney Yin deficiency, which may further limit the external validity of the results, particularly in non-TCM clinical settings. Therefore, the present findings are most directly applicable to perimenopausal women with this specific TCM pattern rather than to all women with perimenopausal symptoms. Nevertheless, the observed improvements in broadly used symptom-based outcomes, including menopausal symptom severity, sleep quality, and quality of life, suggest that the potential benefits of BQG may have relevance beyond the TCM framework, although this remains to be confirmed in broader and more heterogeneous populations. Third, the treatment and follow-up duration was relatively short: participants received BQG or placebo for 4 weeks and were followed for only an additional 4 weeks after treatment completion. Although this design allowed us to capture short-term symptomatic changes and early safety signals, it was insufficient to determine the long-term durability of clinical benefits, the persistence of effects after treatment discontinuation, or the longer-term safety of combined BQG and HRT use. Therefore, the observed between-group differences at week 8 should be interpreted as preliminary evidence of short-term benefit rather than definitive proof of sustained long-term efficacy or safety. Fourth, the placebo contained 3% BQG extract to improve sensory matching and preserve blinding. Although this proportion was far below the therapeutic dose and was intended for placebo simulation rather than therapeutic exposure, a minor biological effect cannot be completely excluded. If present, such an effect would be expected to reduce the observable between-group differences and thus lead to a more conservative estimate of treatment efficacy rather than an overestimation of the benefit of BQG. Further methodological refinement of placebo design may help optimize blinding while minimizing the possibility of residual biological activity in future trials. More broadly, future multicenter studies with larger sample sizes, longer treatment exposure, extended follow-up periods, and mechanistic investigations are warranted to further confirm the long-term efficacy, safety, and clinical applicability of BQG as a complementary treatment for perimenopausal women, including in broader perimenopausal populations not selected according to a specific TCM pattern.

## 5 Conclusion

In this 8-week study, BQG as an adjunct to Femoston was associated with additional short-term improvements in perimenopausal symptoms among women with kidney Yin deficiency, with acceptable tolerability. These findings support the potential role of BQG as a complementary therapy; however, studies with longer treatment and follow-up are needed to confirm the durability of benefit and long-term safety.

## Data Availability

The original contributions presented in the study are included in the article/**Supplementary Material**. Further inquiries can be directed to the corresponding authors.
